# Perceived stress in four inflammatory skin diseases: an analysis of data taken from 7273 adult subjects with acne, atopic dermatitis, psoriasis or hidradenitis suppurativa

**DOI:** 10.1111/jdv.18016

**Published:** 2022-02-28

**Authors:** L. Misery, M. Chesnais, S. Merhand, R. Aubert, M.F. Bru, C. Legrand, H. Raynal, C. Taieb, M.‐A. Richard

**Affiliations:** ^1^ Univ Brest LIEN Brest France; ^2^ Private practice Les Fougerets France; ^3^ Association Française de l’Eczéma Redon France; ^4^ France Psoriasis Paris France; ^5^ Association Française pour la Recherche sur l'Hidrosadénite St Benoit France; ^6^ France Acné Adolescents Adultes Vincennes France; ^7^ Solidarité Verneuil Villeurbane France; ^8^ European Market Maintenance Assessment Fontenay sous‐Bois France; ^9^ CEReSS‐EA 3279 Research Centre in Health Services and Quality of Life Aix Marseille University Dermatology Department University Hospital Timone Marseille France

## Conflicts of interest

None.

## Funding sources

Project funded by European Market Maintenance Assessment with institutional support from Almirall, Leo‐Pharma, Pfizer, Bioderma, Pierre Fabre, Amgen, Sanofi and La Roche Posay.

Editor

Adult acne (AA), atopic dermatitis (AD), psoriasis (P) and hidradenitis suppurativa (HS) are common and chronic, inflammatory skin diseases with an incidence that has been estimated at almost 15% of the adult population in France.^1^ They are often accompanied by increased psychological stress levels.^2^

This observational, cross‐sectional, non‐comparative study conducted by five patient associations in France between October 2020 and February 2021, assessed perceived stress in adults with AA, AD, P or HS, as well as self‐perceived disease severity and quality of life (QoL) in a large population using a digital questionnaire. The questionnaire was distributed directly to patient association members or through social networks. The study complied with local legal requirements for the conduct of this type of study and received ethics committee approval (CPP Ile de France X, 2020‐A01621‐38).

The questionnaire ensured that the target dermatoses had previously been confirmed by a health care professional. Stress was assessed using the validated perceived stress scale (PSS) and QoL using the DLQI.^3^, ^4^ The PSS questionnaire measures the degree to which situations in one's life are considered as stressful. Individual scores on the PSS can range from 0 to 40, with higher scores indicating higher perceived stress. Scores ranging from 0 to 13 indicate low perceived stress, scores from 14 to 26 indicate moderate perceived stress and scores from 27 to 40 indicate high perceived stress. A DLQI score above 10 designates an important or very important impact on the patient’s QoL. Moreover, patients were asked to assess the severity (mild, moderate or severe) of their dermatosis at the time the study was conducted and to confirm if they had been offered psychological support with regards to their dermatosis.

Overall, 7273 subjects participated in this survey; 1605 subjects had AA, 2538 AD, 2329 P and 801 HS. The average age was 40.6 years; 69.25% were women and 54.37% were employed. The self‐assessed disease severity was moderate in 49.73% of subjects.

Stress according to disease is displayed in Table 1. In total, 66.3% of subjects reported stress scores above 27. Results for AA, AD and P showed that the more severe the condition, the higher the perceived stress scores. This could not be observed for HS patients.

**Table 1:** Perceived stress data for acne, atopic dermatitis, psoriasis and hidradenitis suppurativa

	Global population		Subjects with stress expressed as Mild**		Subjects with stress expressed as Moderate**		Subjects with stress expressed as Severe**	
**Adult acne**	**N**	**%**	**N**	**%**	**N**	**%**	**N**	**%**
**Female**	1275	79.44%	768	75.89%	289	88.11%	95	71.43%
<25 years	911	56.76%	46.44%	240	73.17%	69	51.88%	46.44%
26–55 years	633	39.44%	48.52%	82	25.00%	60	45.11%	48.52%
>56 years	61	3.80%	5.04%	6	1.83%	4	3.01%	5.04%
**Perceived stress score** *								
<13	101	8.67%	77	9.27%	13	7.03%	8	7.02%
14–26	244	20.94%	186	22.38%	31	16.76%	18	15.79%
≥27	820	70.39%	568	68.35%	141	76.22%	88	77.19%
**Atopic dermatitis**	**N**	**%**	**N**	**%**	**N**	**%**	**N**	**%**
**Female**	1616	63.67%	699	61.53%	594	61.94%	165	63.22%
<25 years	307	12.10%	117	10.30%	113	11.78%	34	13.03%
26–55 years	1761	69.39%	774	68.13%	684	71.32%	188	72.03%
>56 years	470	18.52%	245	21.57%	162	16.89%	39	14.94%
**Perceived stress score** *								
<13	338	13.60%	239	21.09%	76	7.97%	16	6.35%
14–26	525	21.12%	290	25.60%	173	18.15%	34	13.49%
≥27	1623	65.29%	604	53.31%	704	73.87%	202	80.16%
**Psoriasis**	**N**	**%**	**N**	**%**	**N**	**%**	**N**	**%**
**Female**	1472	63.20%	993	62.14%	224	68.50%	94	64.38%
<25 years	128	5.50%	97	6.07%	17	5.20%	6	4.11%
26–55 years	1265	54.32%	873	54.63%	213	65.14%	93	63.70%
>56 years	936	40.19%	628	39.30%	97	29.66%	47	32.19%
**Perceived stress score** *								
<13	82	4.57%	68	4.85%	11	4.04%	3	2.48%
14–26	524	29.18%	433	30.86%	65	23.90%	26	21.49%
≥27	1190	66.26%	902	64.29%	196	72.06%	92	76.03%
**Hidradenitis suppurativa**	**N**	**%**	**N**	**%**	**N**	**%**	**N**	**%**
**Female**	674	84.14%	82	85.42%	348	89.00%	201	76.72%
<25 years	125	15.61%	10	10.42%	61	15.60%	41	15.65%
26–55 years	635	79.28%	78	81.25%	318	81.33%	203	77.48%
>56 years	41	5.12%	8	8.33%	12	3.07%	18	6.87%
**Perceived stress score** *								
<13	73	17.51%	4	9.76%	35	16.20%	31	22.79%
14–26	124	29.74%	8	19.51%	68	31.48%	43	31.62%
≥27	220	52.76%	29	70.73%	113	52.31%	62	45.59%

* According to the PSS scale.

** According to the patient (evaluation made on a VAS from 0 to 10).

[Correction added on 11 May 2022, after first online publication: In Table 1, there were formatting errors and were corrected in this version.]

While the level of stress was very high in all groups and especially in those patients with severe disease forms, less than 15% had been offered psychological support and, when such aid was proposed, only two patients out of three had accepted it. This finding requires special attention for future patient‐centred care measures to be put in place.

The DLQI score was significantly higher and impacted the QoL of patients with AA, AD and P more, especially when stress scores were above 27, which should encourage health care professionals to develop a different, patient‐centred treatment approach; these results paralleled results observed for perceived stress (Table 2).

**Table 2:** Perceived stress and impact on quality of life

	GLOBAL		Perceived stress score <27		Perceived stress score ≥27		*P*‐value	
	N	Mean or %	N	Mean or %	N	Mean or %	Test Student	Pearson's Chi‐squared test
**Adult acne**								
DLQI (mean score)	1003	7.1	276	4.0	726	8.6	<0.0001	
DLQI >10 * (%)	276	27.5%	27	9.8%	249	34.3%		<0.0001
**Atopic dermatitis**								
DLQI (mean score)	2332	7.2	642	3.6	1623	8.4	<0.0001	
DLQI >10 * (%)	688	29.5%	62	9.7%	626	38.6%		<0.0001
**Psoriasis**								
DLQI (mean score)	1671	7.0	421	4.2	1189	8.0	<0.0001	
DLQI >10 * (%)	478	28.6%	64	13.3%	414	34.8%		<0.0001
**Hidradenitis suppurativa**								
DLQI (mean score)	417	15.9	197	17.9	220	15.8	<0.0001	
DLQI >10 * (%)	309	74.1%	163	82.7%	146	66.4%		0.0001

Not all patients have responded to the entire questionnaire.

[Correction added on 11 May 2022, after first online publication: In Table 2, there were formatting errors and were corrected in this version.]

The main limitations of our study are that the population may be considered unrepresentative, that there was no control group and that the HS population was potentially too small to detect similar results. Moreover, subjects, as members of patient organizations, may suffer more from their disease and could, therefore, be more stressed. Moreover, the gender distribution is not representative, as a majority of women participated in the survey.

Despite these limits and bias, our study shows that in patients with chronic inflammatory skin diseases, psychological stress is an important issue, requiring specific attention and personalized psychological support. Implementing a patient‐centred management in chronic inflammatory skin diseases may reduce psychological stress, and potentially improve treatment adherence and improved treatment outcome.

## Data Availability

The data that support the findings of this study are available from the corresponding author upon reasonable request.
